# Mortality in Swedish patients with Hirschsprung disease

**DOI:** 10.1007/s00383-017-4150-z

**Published:** 2017-09-07

**Authors:** Anna Löf Granström, Tomas Wester

**Affiliations:** 1 Department of Women’s and Children’s Health, Karolinska Institutet, Stockholm, Sweden; 2 Division of Pediatric Surgery, Astrid Lindgren Children’s Hospital, S3:02, Karolinska University Hospital, Solna, 17176 Stockholm, Sweden

**Keywords:** Hirschsprung disease, Mortality, Epidemiology

## Abstract

**Purpose:**

Hirschsprung disease (HSCR) has previously been associated with increased mortality. The aim of this study was to assess mortality in patients with Hirschsprung disease in a population-based cohort.

**Methods:**

This was a nationwide, population-based cohort study. The study exposure was HSCR and the study outcome was death. The cohort included all individuals with HSCR registered in the Swedish National Patient Register between 1964 and 2013 and ten age- and sex-matched controls per patient, randomly selected from the Population Register. Mortality and cause of death were assessed using the Swedish National Causes of Death Register.

**Results:**

The cohort comprised 739 individuals with HSCR (565 male) and 7390 controls (5650 male). Median age of the cohort was 19 years (range 2–49). Twenty-two (3.0%) individuals with HSCR had died at median age 2.5 years (range 0–35) compared to 49 (0.7%) controls at median age 20 years (0–44), *p* < 0.001. Hazard ratio for death in HSCR patients compared to healthy controls was 4.77 (confidence interval (CI) 95% 2.87–7.91), and when adjusted for Down syndrome, the hazard ratio was 3.6 (CI 95% 2.04–6.37).

**Conclusions:**

The mortality rate in the HSCR cohort was 3%, which was higher than in controls also when data were adjusted for Down syndrome.

## Introduction

Hirschsprung disease (HSCR) is a developmental defect of the enteric nervous system caused by incomplete migration, differentiation, and survival of enteric nervous progenitors. The birth prevalence is 1 in 5000 living newborns [1]. HSCR can be a part of a syndrome, most commonly trisomy 21 (Down syndrome). The etiology is still unknown, but HSCR is a multifactorial disease, probably caused by both environmental and genetic factors [2]. Before the era of possible surgical treatment for HSCR, the mortality rate was very high and only patients with short-segment aganglionosis had any chance of survival. Since the surgical procedure became available in the 1950s, the mortality rate has decreased significantly. Postoperative mortality after the Swenson procedure has been reported to be 2.4% between 1947 and 1986 [3]. The mortality in HSCR patients undergoing one-stage transanal pull-through varies between 0 and 2% [4, 5]. Patients with Down syndrome (DS), total colonic aganglionosis (TCA), and Hirschsprung-associated enterocolitis (HAEC) seem to have an increased risk of mortality, as well as patients with anastomotic leakage after the pull-through [3, 6, 7]. HAEC is the most threatening complication of HSCR, since morbidity and mortality are possible outcomes [8]. The pathogenesis remains unknown. HAEC occurs in 5–42% of cases and may develop both before and after surgery for HSCR [9].

The aim of this study was to assess the mortality rate among Swedish patients diagnosed with Hirschsprung disease and to compare the mortality rate with an age- and gender-matched cohort.

## Methods

### Study design and settings

This was a nationwide, population-based cohort study during the observational period 1st of January 1964 to 31st of December 2013. The study exposure was HSCR and the primary study outcome was death. Exposure and outcomes were assessed through linkage between the Swedish National Patient Register and the Swedish National Causes of Death Register. All residents in Sweden get a unique ten-digit personal identification number after birth or immigration, which enables linkage between the national registers.

### Data resources/registers

The Swedish National Patient Register contains prospectively collected information from all hospital admissions in Sweden and is maintained by the Swedish National Board of Health and Welfare. The register was initiated in 1964 and it covers all hospitals in Sweden from 1987. The data include gender, age, geographical data, surgical procedures, date of admission and discharge, and primary and secondary diagnosis. The International Classification of Diseases (ICD) is used to register diagnosis. This classification has been modified over the years: ICD-7 in 1964–1968, ICD-8 in 1969–1986, ICD-9 in 1987–1996, and ICD-10 since 1997. From 2001, data on outpatient specialist care were also included in the register. The most recent validation of the register showed that the diagnoses are valid in 85–95% of the cases [10].

The Swedish National Causes of Death Register is also maintained by the Swedish National Board of Health and Welfare. The register was initiated in 1961 and contains information about all deaths in Swedish citizens since then. Data as cause of death according to ICD classification, date of death, age at death, and place of death are recorded in the register for each death.

### Participants

The cohort was collected from Statistics Sweden and the Swedish National Patient Register. Data on the exposure, HSCR, were collected from the Swedish National Patient Register (ICD-7: 756.31, ICD-8: 751.39, ICD-9: 751D, ICD-10: Q431) during the study period. A total of 1267 individuals with these ICD codes were found. To confirm that they had HSCR and were not misdiagnosed by mistake, each case had to satisfy one of the following inclusion criteria:

1. HSCR as main diagnosis and a surgical intervention number specific for HSCR;
2. admission to a pediatric surgical center at least twice, with a hospital stay of at least 4 days, at least once, and HSCR as main diagnosis for both hospital stays;
3. one long admission (≥4 days) at a pediatric surgical center once and more than one outpatient visit at a pediatric surgical center with HSCR as main diagnosis.

For instance, we wanted to avoid including neonates with suspected HSCR admitted for rectal suction biopsies, where the biopsies turned out to be negative or patients admitted only to a hospital without pediatric surgery.

**Fig. 1:**
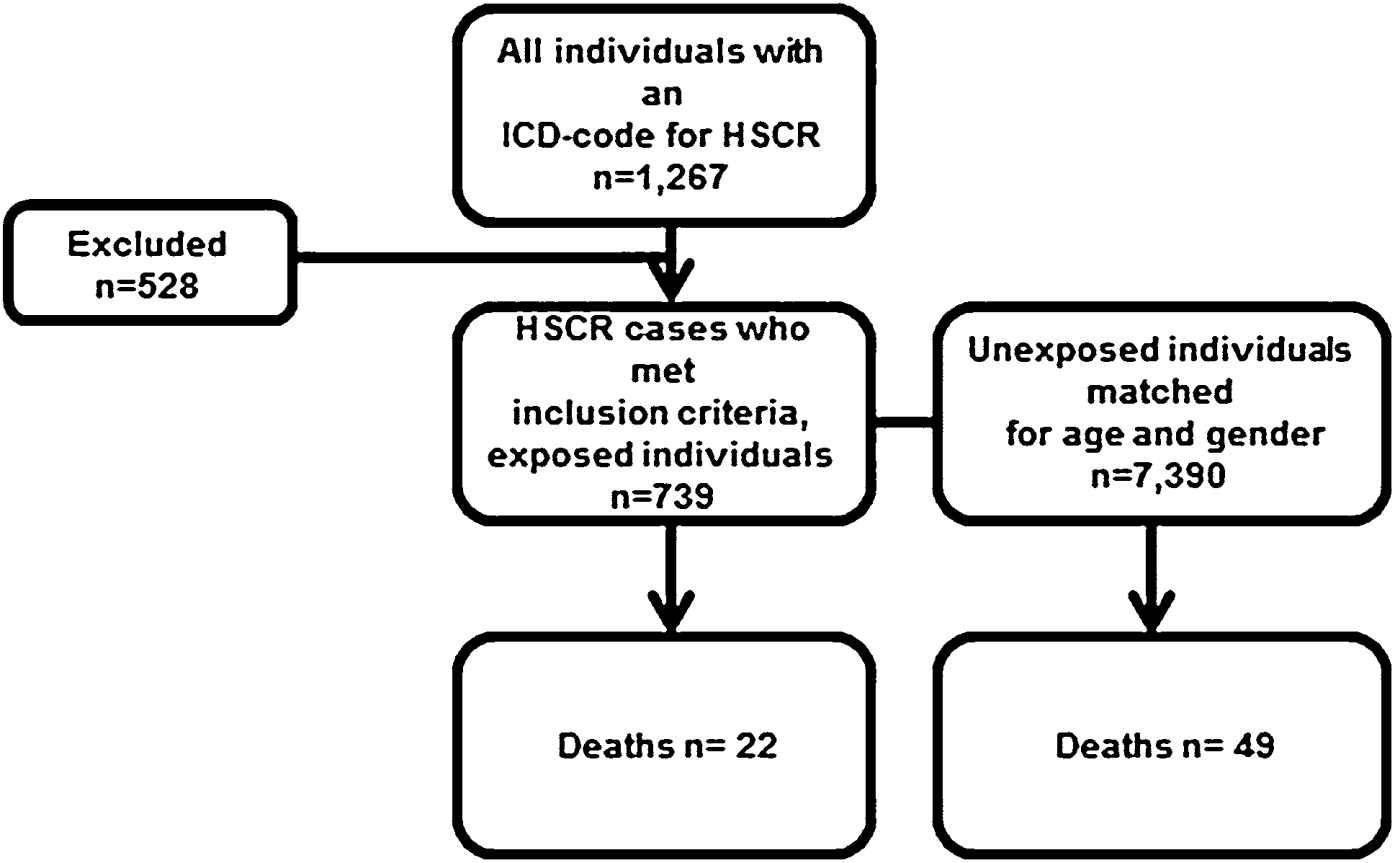
Flow chart for the study

Using these criteria, 528 individuals were excluded, ending up with 739 exposed cases. The unexposed individuals in the cohort were collected from the Swedish National Population Register and comprised ten unexposed individuals for each exposed individual matched for birth year and gender (*n* = 7390) (Fig. 1).

### Variables

The study outcome death was defined as any registration of death in the Swedish National Causes of Death Register. The cause of death was based on ICD classification from the Swedish National Causes of Death Register. HSCR is associated with trisomy 21, which was considered a potential bias. Individuals with Down syndrome were identified in both cohorts in the Swedish National Patient Register (ICD8: 759.3, ICD9: 758A, and ICD10: Q90.0–90.9).

### Statistical analysis

The association between exposed and unexposed individuals was analyzed with R program [11]. Categorical data are presented as frequencies or proportions and analyzed with two-tailed Fisher’s exact test. Numerical data are presented as median and range and two-sided Mann–Whitney *U* test was used for analysis. *p* < 0.05 was considered statistically significant. The Hazard ratio was used for calculations of risk of death and a logistic regression model presented as Odds ratio (OR) and 95% CI was used for calculation of changes in death over time.

### Ethics

The Regional Ethics Review Board in Stockholm approved the study.

## Results

**Fig. 2:**
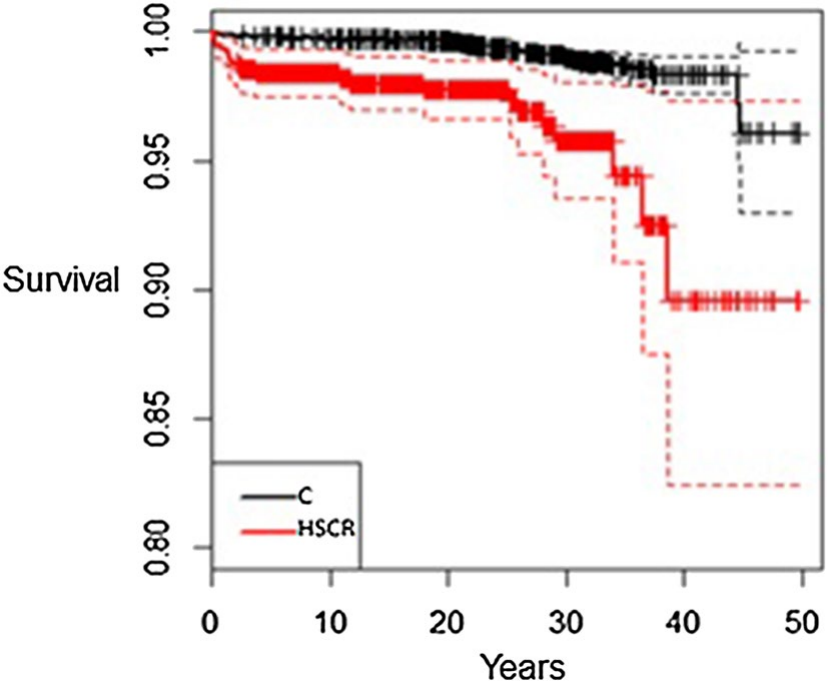
Kaplan–Meier estimate for total survival for the cohort

**Fig. 3:**
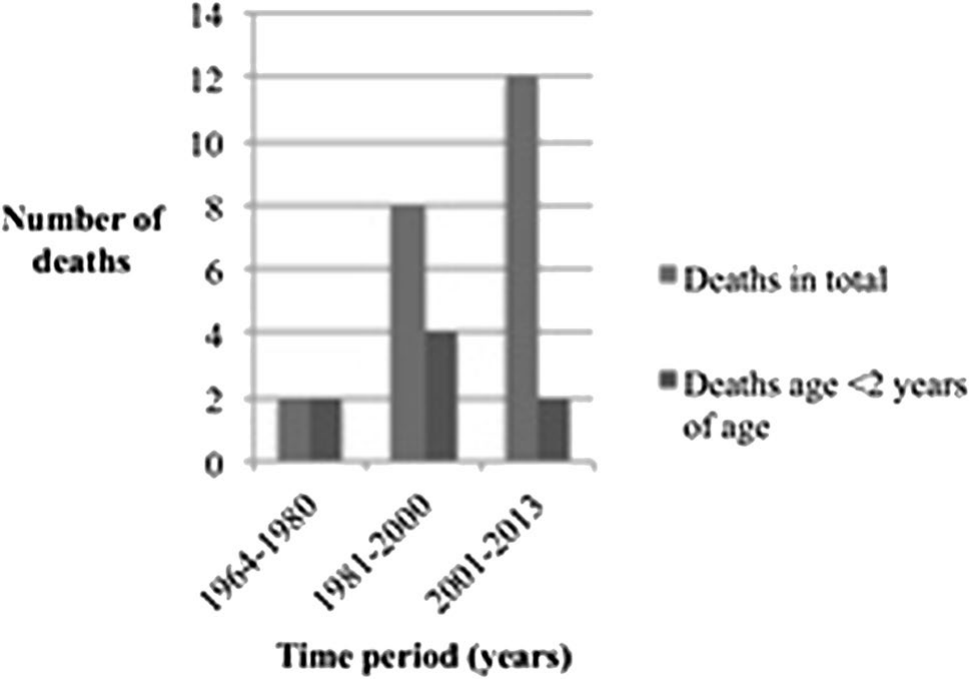
Time for deaths in the HSCR cohort

The cohort comprised 739 individuals with HSCR (565 male) and 7390 controls (5650 male). Median age of the cohort was 19 years (2–49). Twenty-two (3.0%) individuals with HSCR had died at median age 2.5 years (0–35) compared to 49 (0.7%) controls at median age 20 years (0–44), *p* < 0.001. The hazard ratio for death in HSCR patients compared to healthy controls was 4.77 (CI 95% 2.87–7.91), and when adjusted for Down syndrome, the Hazard ratio was 3.6 (CI 95% 2.04–6.37). The Kaplan–Meier analysis showed lower survival rate in the HSCR cohort (Fig. 2). The three most common causes of death among patients with Hirschsprung disease were infections, Hirschsprung disease, and congenital malformations, and in the control group, the three most common causes were suicide, cancer, and trauma. Deaths in the HSCR cohort sorted by decades are presented in Fig. 3. There was no significant decrease in death rates between 1964–1980, 1981–2000 (OR 0.60, CI 95% 0.12–4.16), and 2001–2013 (OR 0.40, CI 95% 0.05–3.34). The mortality data on the HSCR individuals are listed in Table 1.

**Table 1:** Mortality data on HSCR individuals in the cohort

Year of birth	Gender	Trisomy 21	Age at death (years)	Cause of death
1970	F	N	39	Bowel obstruction
1971	M	N	36	Heart failure
1976	M	N	29	HSCR
1977	M	N	12	Drowning
1978	M	Y	34	Budd Chiaris syndrome
1980	F	N	1	Ataxia
1980	M	N	1	HSCR
1981	M	N	28	Epilepsy
1982	M	N	26	Encefalit
1983	F	N	0	Malformations
1983	M	Y	2	Down syndrome
1984	M	N	25	Homicide
1986	M	Y	2	Leukemia
1989	M	Y	1	Malaria
1989	F	N	1	Abdominal infection
1992	M	N	0	HSCR
1993	M	N	18	Immune deficiency
1994	F	N	11	Trauma
1996	M	Y	2	Unknown
2001	M	Y	0	Down syndrome
2007	M	N	4	Unknown
2010	M	N	0	HSCR

## Discussion

### Key results

This is a large national population-based register cohort study, showing that mortality rate was 3% among the HSCR patients. The risk for death was significantly higher in the HSCR cohort compared to the unexposed cohort, but there was no difference in age at death.

### Interpretation

In the literature, mortality among HSCR patients is reported between 0 and 2.4% [3–5]. The follow-up time in these studies varies. Our study shows a mortality rate of 3% in data based on a national register with a long-term median follow-up. Data from another population-based study of patients with HSCR between 1990 and 2008 in the North of England showed that 9% of the children died during their first year of life [13]. In our study, four children died within their first year of life (0.5%) indicating a higher in survival rate in our cohort. As a speculation, this may reflect changes during the latest years as early treatment in patients with suspected HAEC as well as changes in the surgical and anesthetical procedures.

Data have shown that trisomy 21, HAEC, and TCA increase the risk for death, and in this study, the risk for death is still significantly increased although when adjusted for Down syndrome [3, 6, 7]. In addition, assessing the ICD classifications of causes of death indicates a difference between the exposed cohort and the unexposed. For every death, there is at least one ICD classification in the register explaining the main cause of death. Looking at cause of death in the HSCR cohort, four of the patient had HSCR as their main diagnosis. Unfortunately, HAEC does not have an own ICD code. HAEC could potentially have been the cause of death in patient with HSCR registered as cause of death. In this study, we were not able to study if TCA increased the risk for death due to lack of clinical data in the national registers.

### Limitations

This study was based on prospectively collected national register data, which previously shown to have high validity. Since this is a register-based study, no histopathology reports were possible for HSCR diagnose. To reduce the risk for misclassification, specific inclusion criteria were set in advance to identify the exposure of HSCR. This is a limitation of the study, since we may have included patients without HSCR, but also excluded patients with HSCR. One other limitation is that data on HAEC or level of aganglionosis cannot be collected from the registers. Since we know that these factors increase mortality among patients with HSCR, it would have been interesting to include the data in a subanalysis.

The control cohort was randomly selected from Statistics Sweden, reducing the risk for selection bias. To decrease the risk for confounders, the controls were matched for birth year and gender. One other confounder is the fact that HSCR is associated with Down syndrome. Individuals with Down syndrome often have other congenital malformations and have an increased mortality rate [12]. We analyzed unadjusted data and also data adjusted for Down syndrome, and could not show any affect on the mortality rate.

### Generalizability

Being based on a national population-based study, these results are considered highly generalizable. Individuals with HSCR have an increased risk of mortality compared to an unexposed cohort.
